# Efficacy and safety of conventional long acting β2- agonists: systematic review and meta-analysis

**Published:** 2016

**Authors:** Reza Karbasi-Afshar, Jafar Aslani, Mostafa Ghanei

**Affiliations:** 1 Atherosclerosis Research Center, Baqiyatallah University of Medical Sciences, Tehran, Iran.; 2 Chemical Injuries Research Center, Baqiyatallah University of Medical Sciences; Tehran, Iran.

**Keywords:** COPD, β2-Agonists, drug, chronic obstructive pulmonary disease, meta analysis

## Abstract

**Background:**

Chronic obstructive pulmonary disease (COPD) is usually considered one of the leading causes of death worldwide, so finding proper therapeutic strategies for this disease is of high importance. In this meta-analysis, we reviewed the existing literature on the efficacy and safety of conventional long acting beta agonists (LABAs) in COPD patients.

**Methods:**

We searched MEDLINE and Google scholar to identify relevant articles. We limited data to double-blinded randomized controlled trials (RCTs). Data of 14, 832 COPD subjects including 7540 patients under a β_2_ agonist (cases) and 7292 taking placebo (controls) retrieved from 20 randomized controlled trials and were enrolled into this meta-analysis. Evaluated outcomes included overall mortality, exacerbations and tolerance to the drug.

**Results:**

The analysis of survival showed no significant difference between those taking LABAs or placebo (relative risk (RR): 0.945, 95% confidence interval (CI): 0.821-1.088, P=0.432). Exacerbation rate, however, was significantly lower among the cases than among the controls (RR: 0.859, 95%CI: 0.800-0.922, p<0.001). Similar observation was detected in analyzing the rate of drug withdrawal in patients of the two groups with patients under placebo having significantly higher rate of drug discontinuation due to adverse events or disease symptoms (RR:0.821, 95% CI: 0.774-0.871; p<0.007).

**Conclusion:**

In conclusion, we found that the use of conventional LABA therapy in COPD patients is associated with a lower exacerbation rate of the disease as well as higher tolerance to the drug, but no survival advantage is expectable. Substitution of LABAs with new agents is recommended.

Chronic obstructive pulmonary disease (COPD) is considered as one of the leading causes of death worldwide, with about 90% of COPD deaths occurring in the developing world (1), and it is predicted that it will become the third leading cause of death throughout the world by 2030 (2). COPD is a grave and disabling condition that imposes a great deal of either health or financial burden on the patient and society. In this disease, lung function deteriorates through years with increasing respiratory complaints (including dyspnea, cough and sputum production). As the disease progresses, acute exacerbations become more common, especially in later stages that do not only affect patients’ daily activities and well-being (3), but also can predict higher mortality (4).

Pharmacotherapy is a major therapeutic approach to COPD patients which consists of prescription of several agents including bronchodilators, such as β_2_-agonists and inhaled corticosteroids. Due to our purpose in the treatment of COPD which is a better management of patients’ symptoms, reduce exacerbations and prevent death rate, we need to know how much our treatment strategies are safe and efficient. 

The long-acting β_2_-agonists formoterol and salmeterol have long been used to improve lung function and reduce symptoms and improve outcome in COPD patients. There are studies both in favor of using these agents in the mentioned patients and against them, but to have the most comprehensive view on the topic of efficacy and safety of these conventional β_2_-agonists, there is a need to conduct systematic review of the randomized controlled trials published on this issue. For the same reason, we performed this study to review the existing literature and to conduct a meta-analysis to find the efficacy and safety of conventional β_2_ agonists in COPD patients.

## Methods

To conduct our systematic review, the primary search was done using the keywords "salmeterol" and “COPD” within the time-span of 1990-2013. A repeat of the search using “formoterol” instead of “randomized controlled trial” was performed to expand the included studies. Again, the literature search was repeated using the terms “long-acting beta agonist” and “efficacy” or “safety” or “exacerbation” or “withdrawal” or “randomized controlled trial”. A literature search was performed using Pubmed database, which we believe provided relatively the largest published data of the most relevant studies in the field of pulmonary diseases. We also tried to boost our search on citations of the found articles to find potential reports which were not indexed in Pubmed or retrieved through Pubmed search.

In our search, overall, 892 studies were found in the literature search in Pubmed database using the mentioned keywords. Then found titles of the studies were screened to find appropriate studies associated with our systematic review, and randomized controlled trials. Finally, 20 randomized controlled trials investigating the efficacy and safety of salmeterol or formoterol on the disease course, drug tolerance and survival of COPD patients were enrolled into the Meta-analyses (table 1) (5-24). The analysis was performed in three major study variables: exacerbations, drug withdrawal and patient’s survival. 

**Statistical analysis: **The meta-analysis has been performed using software Stata v.9.0 (Stata corp, TX, USA). 

**Table 1:** The included randomized controlled trials

**trial**	**Trial author (year)**	**Reference no.**	**Year**	**Case group (n)**	**Control (n)**	**Beta agonist**
1	W. Szafranski et al. (2003)	5	2003	201	205	Formoterol
2	Peter Calverley et al. (2003)	6	2003	372	361	Salmeterol
3	Peter M.A. Calverley et al. (2007)	7	2007	1521	1524	Salmeterol
4	Nicola A. Hanania et al. (2003)	8	2003	177	185	Salmeterol
5	P.M. Calverley et al. (2003)	9	2003	255	256	Formoterol
6	Christine R Jenkins et al. (2009)	10	2009	1521	1524	Salmeterol
7	Andrea Rossi et al. (2002)	11	2002	214	220	Formoterol
8	M. Wadbo et al. (2002)	12	2002	61	60	Formoterol
9	Donald A. Mahler et al. (1999)	13	1999	135	143	Salmeterol
10	Donald A. Mahler et al. (2002)	14	2002	160	181	Salmeterol
11	Kenneth R Chapman et al. (2002)	15	2002	201	207	Salmeterol
12	Ronald dahl et al. (2001)	16	2001	194	200	Formoterol
13	Ronald Dahl et al. (2010)	17	2010	434	432	Formoterol
14	V Brusasco et al. (2003)	18	2003	405	400	Salmeterol
15	James F. Donohue et al. (2002)	19	2002	213	201	Salmeterol
16	G. Boyd et al. (1997)	20	1997	229	227	Salmeterol
17	RudolfA. Baumgartner et al. (2007)	21	2007	144	143	Salmeterol
18	B. CELLI et al. (2003)	22	2003	554	271	Salmeterol
19	Malcolm Campbell et al. (2005)	23	2005	215	217	Formoterol
20	O. Kornmann et al. (2011)	24	2011	334	335	Salmeterol

## Results

Data of 14, 832 COPD subjects including 7540 patients under a β_2_ agonist and 7292 patients taking placebo were retrieved from 20 randomized controlled trials and were enrolled into this meta-analysis. From the 7540 COPD patients under a β_2_ agonist, 1574 were taking formoterol and the remaining 5966 patients were under salmeterol therapy. 

**Figure 1:**
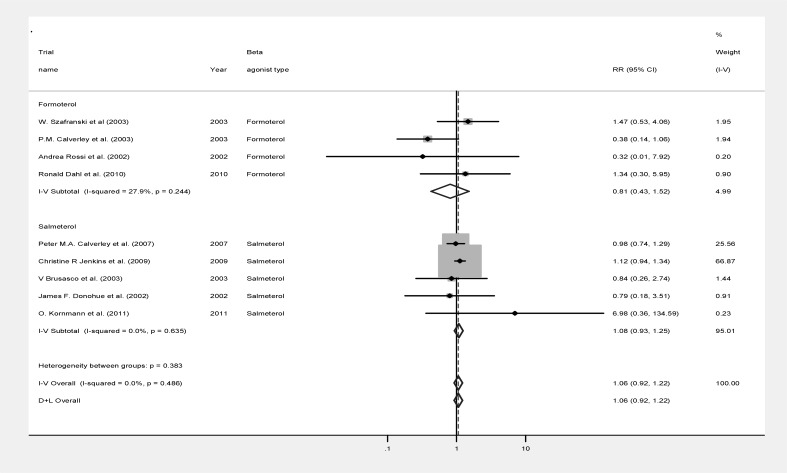
Forest plot of meta-analysis of 9 randomized controlled trials investigating survival of patients using conventional β_2_ agonists compared to placebo

**Analysis of survival:** Figure 1 summarizes the data of the analysis. Analysis of survival of patients in the two groups showed no significant difference between those taking beta agonists or placebo (relative risk (RR): 0.945, 95% confidence interval (CI): 0.821-1.088, p=0.432, z=0.79; figure 1). 

Reanalysis of data including only the patients receiving one of the beta-agonists did not change the results. No significant heterogeneity has been observed among the survival data of the included studies, indicating a high reliability value for the analysis (P=0.486; heterogeneity χ^2^=7.48 (d.f.=8) I-squared (variation in RR attributable to heterogeneity) = 0.0%). 

**Figure 2:**
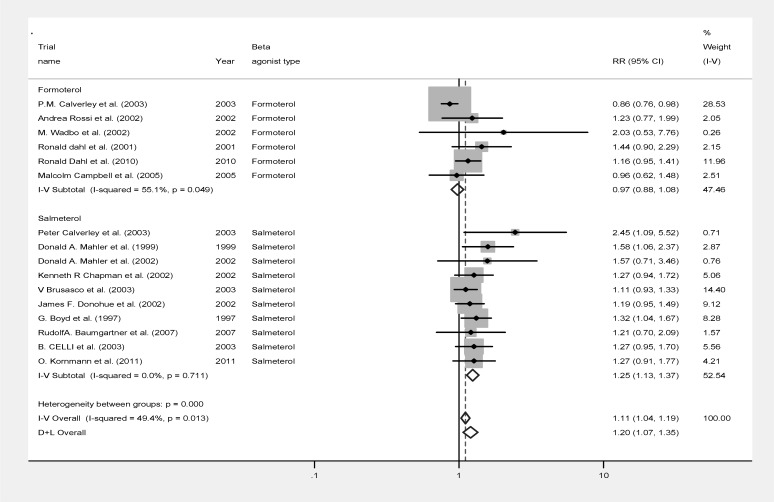
Forest plot of meta-analysis of 16 randomized controlled trials investigating disease exacerbations of COPD patients using conventional β_2_ agonists compared to placebo

**Exacerbations:** Figure 2 summarizes the data of the analysis. Analysis of the rates of the patients experiencing exacerbation episodes within the trial period, however, showed that COPD patients taking β_2_ agonist were significantly less likely to develop an exacerbation episode (RR: 0.859, 95%CI: 0.800-0.922, p<0.001, z=4.19; figure 2). The heterogeneity of the included studies in the exacerbation rate was significantly high (p=0.007, Heterogeneity χ^2^ =31.62 (d.f.=15) I-squared= 52.6%). However, reanalysis of data censoring data of any individual study did not change the significant effect of beta agonists on exacerbation rates, suggesting that none of the studies had such a high magnitude on the analysis individually that was able to skew the results of analysis of the overall studies. 

**Figure 3:**
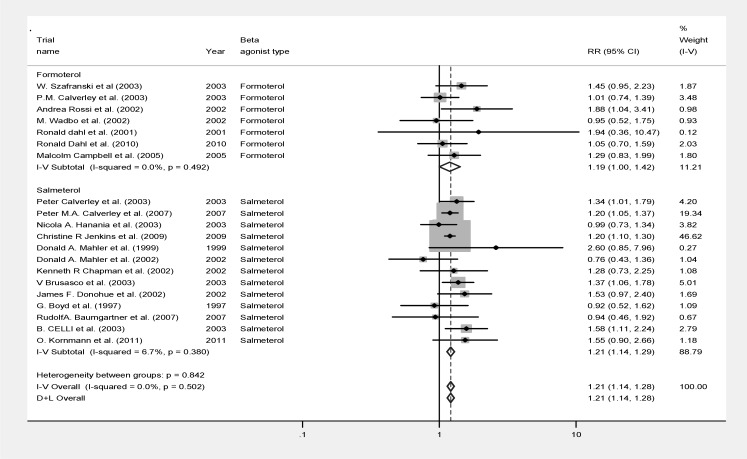
Forest plot of meta-analysis of 20 randomized controlled trials investigating symptom-related treatment withdrawal of COPD patients using conventional β_2_ agonists compared to placebo

**Tolerance to therapy:** Figure 3 summarizes the data of the analysis. Similar observation was detected in analyzing the rate of drug withdrawal in patients of the two groups with patients under placebo having significantly higher rate of drug discontinuation due to adverse events or disease symptoms (RR:0.821, 95% CI: 0.774-0.871; p<0.007, z= 6.52; figure 3). Like what we observed in the analysis of survival, the heterogeneity rate was not significantly high for tolerance to the therapy (P=0.5, heterogeneity χ^2^ =18.34 (d.f.=19) I-squared=0%). 

## Discussion

Despite the fervent debate over the use of long-acting β2 agonists (LABAs) in the treatment of COPD (25), these agents still play a central role in the management of the disease, and are usually considered an inevitable part of treatment regimen in COPD in the majority of clinics (26). In vitro studies have demonstrated that LABAs can boost the Th2 inflammatory pathway by inhibiting interleukin (IL)-12 and interferon (IFN)-γ (27). In vivo, most studies have suggested that β2-agonists increase airway hyper-responsiveness (28). On the other hand, in clinical trials, there are controversial data on the safety and effectiveness of conventional LABAs on the symptoms and outcome of patients with COPD. This urged us to make some comprehensive search study of the current literature so we can reach to a reliable conclusion on the matter based on all the valuable data coming from randomized controlled trials from the literature. 

In this meta-analysis, we showed that conventional LABAs have no survival advantage for COPD patients. Similar findings were reported by a previous meta-analysis, except that they had compared survival effects of inhaled LABAs with corticosteroids (29); while in the current study, we compared it to the placebo which we believe will reveal more fundamental evidence from potential survival effects of LABAs on COPD patients. Our data suggest that LABAs do not only have significantly lesser survival effects than inhaled steroids, but also, it seemed that no outcome effect is expected to be achieved through them. On the other hand, as it has been shown later in the current study, LABAs can improve some of the very important aspects of the disease therapy like alterations in exacerbation rates and good tolerance to the treatment. 

These findings may promote one to presume some survival benefits for LABAs as well. But this discrepancy might be explainable in part with a reported increased risk for adverse events associated with therapy with LABAs in COPD patients (30). LABAs may have adverse cardiovascular effects, deteriorating cardiovascular health in COPD patients with high predisposition to concomitant cardiac disorders (31); and this necessitates caution in administering this family of agents to COPD patients with simultaneous cardiovascular disease (32). Another explanation was provided by TORCH trial that clearly demonstrated long-term use of LABAs for a period of three years was associated with a lower risk of mortality, as compared to placebo (7). Putting together, in short term use of LABAs in COPD patients, no survival advantage is expectable, while it is possible that if patients are controlled for concomitant cardiovascular diseases, and they use LABAs for long-term periods, drug administration shows some life advantages in them. In fact, some new evidence has come to the literature suggesting survival benefits of using cardioselective β_1_ blockers in COPD patients (33). Thus, we recommend future studies to be conducted prescribing cardioselective β_1_ blockers simultaneous to LABAs to evaluate whether this combitation can promise some survival advantage in this patient population. No need to remind that these studies should strongly adhere to the ethical measures to provide their participants with the highest possible safety and support. 

An interesting finding of the current study which we believe is more novel than the remaining is the higher tolerability of therapy with LABAs than the placebo. This finding is of some value and suggests that using LABAs is not quite worthless, and any have some relieving effects on COPD symptoms. However, the lack of strong evidence for survival benefit for LABAs puts them on competition with anticholinergic agents including ipratropium bromide inhaler. 

The lesser rate of COPD exacerbations in patients under therapy with LABAs is another significant finding of this study. It has been well demonstrated that exacerbation episodes are associated with significant higher rates of either short- or long-term survival (34). Only in-hospital mortality of COPD patients admitted with disease exacerbation has been reportedly over 8% (35, 36). Longer term outcome of acute exacerbation of COPD was also high with up to about 50% mortality rate during the first two years post hospitalization (37). The number of exacerbations experienced by each patient was also a determinant of survival (38). Thus, potential survival advantages which may be expected from LABAs in COPD patients are probably compromised by its cardiovascular burden, leaving no significant survival benefit for these drugs. This finding promotes us to try to substitute these highly commonly used agents with other agents, which provide similar symptomatic advantages while having more cost-effectiveness and less side effects, and they can be more available to a larger patient population. 

This study has some limitations. Most importantly, due to a shortage in the number of studies evaluating long-term survival effects of therapy with conventional LABAs in COPD patients, we were not able to analyze this issue. Moreover, censoring the cardiovascular side effects of LABAs from the analysis was not possible. To sum it up, evidence does not suggest any significant survival effect for LABAs in COPD patients, and we recommend to substitute agents of this group with new groups of drugs with more cost- effective values, and/or less side effects. Newly introduced agents which may suggest survival benefit should also be considered for future randomized trials. In conclusion, we found that using conventional LABA therapy in COPD patients is associated with a lower exacerbation rate of the disease as well as higher tolerance to the drug; but no survival advantage can be expected from them. Future studies with more controlled conditions and longer follow-up periods are recommended.
